# Support for Children With Neurodevelopmental Disorders: A Qualitative Study Based on Interviews With School Nurse-Teachers

**DOI:** 10.7759/cureus.74369

**Published:** 2024-11-24

**Authors:** Satoko Yamasaki, Hiromi Kawasaki

**Affiliations:** 1 Department of Health Science, Graduate School of Biomedical and Health Sciences, Hiroshima University, Hiroshima, JPN

**Keywords:** attention-deficit/hyperactivity disorder (adhd), autism spectrum disorder (asd), children, learning disabilities (ld), neurodevelopmental disorders, school nurse teachers, support, yogo teachers

## Abstract

The role of school nurse-teachers (SNTs) in supporting children with neurodevelopmental disorders (CNDs) in compulsory education schools has not been clarified. This study aimed to explore how these professionals manage challenges and provide tailored care for CNDs in such settings. We conducted a qualitative analysis of semi-structured interviews with experienced SNTs. The findings indicate that SNTs employ various strategies, including making environmental adjustments for children with sensory hypersensitivity, communicating clearly according to individual traits, and collaborating with other educators and parents to address the difficulties faced by CNDs. These approaches should be integrated into routine SNT practices to support children’s health and improve their quality of life.

## Introduction

Autism spectrum disorder (ASD), attention-deficit/hyperactivity disorder (ADHD), and learning disabilities (LD) are classified as neurodevelopmental disorders (NDs). ASD is characterized by fixed, rigid behavior and social difficulties, whereas ADHD involves attention difficulties, impulsivity, and hyperactivity [1]. A systematic review reported a global ASD prevalence of 0.6% [2], with approximately 5% of children estimated to have ADHD [3]. In the United States, approximately 2.7% of children aged eight years are estimated to have ASD [4]. In Japan, a survey conducted from 2013 to 2016 reported a cumulative ASD incidence of 3.22% in children aged five years. Of these children, 11.5% had ASD alone, while 88.5% had at least one other ND [5]. Furthermore, a nationwide survey in 2022 reported that 8.8% of students in regular classes in public elementary and junior high schools required special support [6]. This represents an increase from 6.5% in a 2012 survey, indicating a rising trend over the past 10 years. Although the data were entered by classroom teachers and did not necessarily represent students medically diagnosed with NDs, they suggested that a growing proportion of children require special educational support.

Schools are also required to establish appropriate support systems to address the needs of individuals with NDs. In schools, support for children with NDs (CNDs) often involves collaboration between homeroom teachers and school nurse-teachers (SNTs) [7]. Most schools in Japan have a school infirmary staffed by one or more SNTs rather than school nurses. SNTs are responsible for supporting children’s growth and development through health education and services rooted in the principles of health promotion [8]. Although some SNTs hold nursing licenses, their role is primarily educational rather than medical. Their duties include providing first aid, daily health monitoring, health education, health counseling, coordination of school-wide health examinations, and maintaining environmental hygiene in schools.

CNDs, who often require frequent hospital visits, have frequent interactions with SNTs [9]. However, some SNTs report feeling burdened by the demands of supporting CNDs [10]. Approaches employed by SNTs to support CNDs in compulsory education schools have not yet been clarified. Therefore, this study aimed to identify the strategies used by SNTs to support CNDs in Japanese compulsory education schools. Clarifying these approaches may offer valuable insights for supporting CNDs in school, medical, and societal contexts, ultimately improving their quality of life (QOL).

This article was previously presented as a meeting abstract at the 27th East Asian Forum of Nursing Scholars on March 7, 2024.

## Materials and methods

Design

We conducted a qualitative study to explore the support provided by SNTs to CNDs in Japan, focusing on the specific approaches SNTs take when interacting with CNDs. Given that ND diagnoses such as ADHD and ASD overlap in some cases [5,11], we did not limit the students targeted in this study to specific diagnostic labels. Additionally, in Japan, many children remain undiagnosed until they enter elementary school [12]. Furthermore, not all students receive clear diagnostic labels, and some remain in a gray zone [13]. Therefore, in this study, we refer to children requiring special support, including those suspected of having ND, as CNDs.

Participants

Participants were SNTs with a minimum of 15 years of experience in Japan, ensuring sufficient accumulation of experience in supporting CNDs. We recruited participants from Prefectures A and B using the snowball sampling method. Recruitment continued until data saturation was reached, resulting in the inclusion of seven SNTs in the study.

Data collection

Data were collected through interviews conducted between April and August 2023. Participants were fully informed before the interviews about the study’s purpose, methods, voluntary participation, and confidentiality, both orally and in writing, to obtain their consent. The semi-structured interviews were conducted, either face-to-face or online, using an interview guide designed to elicit situations in which SNTs supported CNDs, as well as their intentions and specific support approaches (Table 1). To ensure the appropriateness of the interview questions, we conducted one pilot interview and did not change the questions following the pilot. After confirming the appropriateness of the interview guide, we conducted the main study. Interviews were conducted in quiet, private rooms, either within the participants’ schools at our university or online, with audio recordings made after receiving permission from the participants. At the end of the interview, a gift card worth 2,000 yen (12.5 US dollars) was given to each participant. Interview durations ranged between 35 to 67 minutes (median, 52 minutes) and were continued until thematic saturation was reached [14].

**Table 1:** Structured Interview Guide CNDs: children with neurodevelopmental disorders, ND: neurodevelopmental disorders.

Domains	Questions
I. Education and knowledge to support CNDs	Have you ever attended a lecture on ND or supported a CND?
II. Examples of support for CNDs - successful approaches and the reasons for adopting them	Can you share a specific case where you successfully supported a student suspected of having ND? What approach did you adopt in that case? Why did you adopt that approach?
III. Examples of support for CNDs - approaches to difficult cases and the reasons for adopting them	Can you describe the most difficult case you have experienced while supporting students with ND (including those in the “gray zone” or those suspected of having ND)? What approach did you adopt in that case? Why did you adopt that approach?
IV. Other stories	Are there any other cases you have experienced? Is there anything else that the interviewer may have missed asking about?

Analysis

After removing personal information, we transcribed the audio recordings verbatim and created narrative records, which were then qualitatively analyzed [15]. Two researchers independently coded the interview data, creating codes by condensing the semantic content. The codes were then compared based on differences and similarities, and themes and sub-themes for each semantic segment were identified. The research team engaged in repeated discussions and reviews until a consensus was reached. Additional member checks were performed during the final coding stage to enhance the reliability of the results. Based on feedback from one SNT with >15 years of experience and one researcher with extensive experience in qualitative research who conducted the reevaluation, we improved the themes and sub-themes.

Ethical consideration

This study was conducted in accordance with the guidelines of the Declaration of Helsinki and was approved by the Hiroshima University Epidemiological Research Ethics Review Committee (Approval No.: E2022-0300). Participants were informed verbally and in writing that non-participation would not result in any disadvantages, and informed consent was obtained from all participants.

## Results

Table 2 presents the demographic characteristics of the seven participants. The mean length of work experience as an SNT was 25 years (range, 16-36 years). All participants had worked in elementary schools, and three had experiences in junior high schools (multiple responses were allowed).

**Table 2:** Participant Demographics

Characteristic	N=7, n(%)
Age (years)	
30–39	3 (42.9%)
40–49	0 (0%)
≥50	4 (57.1%)
Sex	
Female	7 (100%)
School nursing experience (years)	
10–19	3 (42.8%)
20–29	1 (14.4%)
≥30	3 (42.8%)
School level of school nursing experience	
Elementary	7 (100%)
Middle/Junior	3 (42.9%)
High school	0 (0%)
Type of school	
Public	7 (100%)
Special education coordinator experience	
Yes	2 (28.6%)
No	5 (71.4%)
Experience with special needs education lectures	
Have taken a lecture on knowledge	7 (100%)
Have taken a lecture on how to support	4 (57.1%)

Content analysis identified 6 themes and 23 sub-themes, revealing SNT approaches to support CNDs (Table 3). 

**Table 3:** School Nurse-Teachers’ Support Approaches for Children With Neurodevelopmental Disorders

Themes	Sub-Themes	Participant Verbatim
1. Providing support to reduce difficulties associated with ND traits	Making environmental arrangements for children with sensory hypersensitivity	“Children with sensory hypersensitivity may dislike the smell of disinfectant; therefore, I ventilate the room frequently.”
	Helping children engage smoothly in activities	“I keep my instructions brief and straightforward. Sometimes instructions like ‘Do it within the count of five’ help them move quickly.’’
	Promoting cool down when a panic episode occurs	“I wait until they calm down because talking to them in an excited state will not get through. I make environmental arrangements such as removing objects that may be dangerous if they bump into them.”
2. Assessing children’s health status through effective interviews and guidance	Providing advanced guidance for children to simulate health examinations	“Some children are afraid of nasal and aural speculums; therefore, I show them these instruments in advance, let them touch them, and reassure them that they do not hurt. I provide visual support by marking where to stand and exit.”
	Conducting effective interviews to make it easier for children to express their symptoms during first aid	“For children who have difficulty communicating verbally, I confirm the situation by drawing pictures together with them.”
	Providing explanations that are convincing to children	“When using metaphors or analogies, I consider their tendency to take things literally.” “Visual information often helps them grasp things easily.”
3. Promoting individual children’s development and adaptation to school life	Promoting children’s social skills	“I teach about personal space, and socially unacceptable behaviors in everyday life.”
	Promoting relationship-building with other children	“For children who have difficulty building relationships with others, I provide support by asking them to create school infirmary postings so that they can make friends with other children in a natural manner.”
	Making reasonable accommodations within set rules	“Even children requiring special support are not allowed to do whatever they want in the infirmary. They still need to attend classes. I maintain clear boundaries.”
	Promoting self-affirmation through support	“I notice and acknowledge their good points.” “I assign tasks that align with the strengths of children with ND traits.”
4. Becoming a person with whom children feel safe to engage	Making efforts to become a familiar and reassuring presence	“To make it easier for them to consult with me when they are in trouble, I keep regular communication with them.”
	Maintaining honesty to avoid losing children’s trust	“It is important to establish a good relationship at the beginning because children with specific developmental traits have a zero/hundred mindset, and once they form an impression of someone, it is difficult to change.” “Keeping promises usually earns their trust.”
	Approaching without preconceived notions, showing receptive behavior and empathetic understanding toward children’s struggles	“I listen receptively and attentively to children who visit the infirmary frequently, without holding any preconceived ideas.” “I try to understand their loneliness and make sure they feel supported and not left alone.”
	Providing reassurance	“Some children are afraid of health examinations. Offering small words of encouragement like ‘You have done well’ helps ease their fear”
	Leading to continuous support	“When sending children back to class after visiting the infirmary, I say to them ‘If it gets tough, feel free to come back’ to ensure support is not interrupted.”
5. Understanding children’s backgrounds that may not be obvious	Observing each child’s situation and behavior to detect any changes in CNDs	“I carefully assess pain levels in children with sensory hyposensitivity.” “It is important to not just take their words like ‘I am fine’ at face value, but also consider their tone and other factors by talking to them.”
	Understanding children’s emotions and drawing out their true feelings	“It is hard for children to express their thoughts and feelings, and the more difficult their situation is, the harder it is for them to say ‘Listen to me, teacher’. This is why I try to find clues in casual conversations”
	Actively seeking information to understand children better	“I assess existing survey results about children to understand their traits before deciding on optimal approaches for them.”
6. Collaborating to provide consistent support	Sharing information about children with teaching staff	“I inform homeroom teachers about not only injuries and illnesses but also unusual behaviors of children who visit the infirmary, no matter whether they are first-time or frequent visitors.”
	Participating in multidisciplinary discussions on support policies	“It is important to understand clearly the difficulties faced by children and what works well for them; therefore, the entire school needs to discuss optimal approaches.” “I should not carry the burden alone. I consult with principal as well”
	Providing consistent support approaches based on a shared understanding among teaching staff	“If collaboration among teaching staff is insufficient, some teachers’ approaches may result in discomfort for children. Therefore, we aimed at a shared understanding and consistent approaches among teaching staff.”
	Sharing information about children with their parents regularly to establish collaborative relationships with them	“I think they find it easy to rely on me because I often tell parents to feel free to reach out whenever they need help. If they trust me, things will go well. That is why relationship-building is so important.”
	Connecting children to specialists and hospitals according to their traits and difficulties	“We collaborate with doctors in medical institutions. Sometimes I accompany parents to listen to specialists’ advice.”

Theme 1: providing support to reduce difficulties associated with ND traits

SNTs supported CNDs in reducing difficulties associated with ND traits, such as sensory hypersensitivity, restricted and repetitive behaviors, obsessions, and uncontrollable panic episodes. For CNDs with sensory hypersensitivity, such as sensitivity to smells and light, SNTs made environmental arrangements, such as frequent ventilation and softer lighting, in the school infirmary. SNTs ensured that the children’s restricted and repetitive behaviors and obsessions did not interfere with various activities in school education, such as sports events, committee activities, and school lunches. For children with concurrent processing difficulties, SNTs facilitated smoother activity engagement by providing straightforward instructions rather than multitasking. Additionally, SNTs considered effective verbal instructions to help CNDs switch tasks or thoughts more smoothly. Furthermore, SNTs anticipated situations in which panic might occur and took preventive measures. SNTs would explain the state of the injury to the CNDs when giving first aid, but they were very selective about the information they conveyed because the CNDs might panic if they heard things like “possible broken bones” or “you need to go to the hospital to have the wound stitched.” Moreover, when CNDs were injured, the SNT predicted that saying, "It hurts, doesn’t it?" could lead to extra pain and panic. When the SNTs gave first aid, they would often say to other children, “This hurt, didn’t it?” and expressed sympathy for the pain, but they did not say this when calming the CNDs. In addition, when CNDs experience panic in the classroom, the homeroom teacher may bring them to a school infirmary to calm down. At that time, the SNT continued to observe them until they calmed down. At the same time, they helped the CNDs calm down smoothly and safely by keeping dangerous objects away and creating a space where the CNDs could be alone by partitioning the room with curtains.

Theme 2: assessing children’s health status through effective interviews and guidance

SNTs assessed and supported CND’s health through advanced guidance on health examinations, effective interviews during first aid, and clear explanations. Health examinations at school include height and weight measurements, vision and hearing examinations, otorhinolaryngological examinations, dental examinations, and electrocardiography. Before the health examinations were conducted, the SNTs explained the purpose of the examinations and how to undergo them in a way that made sense to the CNDs. For example, as some CNDs have difficulty understanding auditory information, the explanation was provided using easy-to-understand visual materials, such as pictures, photos, and videos. In addition, some CNDs are uncomfortable with examinations in new situations or with instruments; therefore, roleplay is used to familiarize children with examinations and equipment. When CNDs receive their health examinations at school, they wait in line and move to their respective examination sites. The positions of the lines were marked on the floor, and arrows were placed on the wall to visually indicate the positions of the lines so that the movement would be smooth. Some CNDs are hyperactive, so we moved them with books so that they could read their favorite books while waiting and prepared chairs for CNDs who had trouble standing and waiting. Accommodations were also made so the children could wait and move comfortably during examinations. In addition, during the health interviews, SNTs used nonverbal communication and effective interviews to make it easier for CNDs to express their symptoms. For example, when eliciting information from CNDs during first aid about the situation at the time of injury, SNTs sought nonverbal communication methods, such as drawing pictures with them to confirm the situation if they had difficulty with verbal expression or using graphs and face scales when confirming the degree of pain. CNDs who had difficulty filling out the medical questionnaire were asked to answer verbally, and SNTs wrote their answers on their behalf.

Theme 3: promoting individual children’s development and adaptation to school life

This theme shows that SNTs supported CNDs by focusing on their social skills, self-affirmation, and relationship-building with other children to promote their adaptation to school life and development. SNTs promote children’s social skills through interactions at the school infirmary. When the CNDs panicked and hit things, the SNTs were encouraged to reflect on the situation while cleaning up the broken items with them, rather than cleaning them up alone. SNTs were also mindful of improving children’s verbal expressions and guided them toward understanding social norms and unspoken rules. SNTs corrected and taught CNDs how to use words that they incorrectly perceived as being used by CNDs, such as during medical interviews. SNTs taught social distance to CNDs who suddenly entered their personal space using concrete objects such as arm length. Through interactions in the school infirmary, SNTs connected CNDs to other children to promote relationship building. When CNDs who had difficulty making friends came to the infirmary, the SNT asked them, along with other children, to make posters to put up in the infirmary. By creating them together, they helped them naturally become friends. Furthermore, SNTs aimed to promote CND’s self-affirmation through support. They provided step-by-step guidance without demanding immediate results, allowed the children to set goals, and acknowledged and praised them, as well as the process. SNTs guided the children toward becoming aware of their own positive qualities while avoiding hurting their pride when providing guidance. In addition, the SNT, in supporting the CNDs, did not allow them to do whatever they wanted but set certain rules, such as “You must tell your homeroom teacher before coming to the school infirmary,” and “Rest in the infirmary for no more than one hour.”

Theme 4: becoming a person with whom children feel safe to engage

This theme summarizes the approaches adopted by SNTs to become people with whom CNDs feel safe to engage. As children who struggle with new experiences, SNTs fostered regular communication and created an approachable atmosphere to become a familiar and reassuring presence. SNTs made efforts to become familiar not only when the children visited the school infirmary but also regularly. To make it easier for children to approach them, SNTs also adjusted their tone of voice to be softer and more welcoming. Additionally, as some children find it challenging to change their perceptions once they are formed, SNTs placed great importance on the first encounter, keeping promises, and maintaining honesty to avoid losing trust. SNTs also approached CNDs without preconceived notions, showing receptive behavior and empathetic understanding of their struggles and providing reassurance. SNT applied these approaches consistently, tracking and recording progress to ensure continuous support.

Theme 5: understanding children’s backgrounds that may not be easily apparent

SNTs actively sought information to understand children better by reading handover documents sharing information from other teachers and parents and observing CNDs in their daily lives and participating in training sessions. Apart from encounters in the school infirmary, SNT made efforts to notice any changes in CNDs by, for example, greeting the CND at the school gate in the morning and patrolling the classrooms to keep track of the CND’s usual appearance. The SNT did not simply accept the CNDs’ words “I’m fine” but made a careful assessment, taking into consideration a variety of factors, including tone of voice and gaze. When providing first aid, SNTs carefully assessed CNDs, especially those with sensory hyposensitivity who may perceive pain less intensely. In addition, SNT did not scold without asking why and recognized that even CNDs’ problematic behaviors could have underlying meanings, and they worked to understand children’s emotions. Concerning children who have difficulty with verbal expression, an SNT stated “Some children cannot say in words what is troubling them; therefore, I consider that even problematic behaviors have some underlying meanings,” revealing that SNTs are mindful of understanding these children’s emotions and seek out ways to uncover their true feelings through casual conversations.

Theme 6: collaborating to provide consistent support

This theme shows the process of collaborating to provide consistent support. At in-school meetings, CNDs’ difficulties and support policies were discussed by a multidisciplinary team, including the principal, vice-principal, homeroom teacher, manager in charge of student guidance, school counselors, school social workers, and SNTs. SNTs shared information about the children who visited the school infirmary with homeroom teachers and school managers. They communicated and shared information with the teaching staff daily, not only during in-school meetings. SNTs also focused on building relationships with parents by regularly updating them regarding their child’s condition and actively communicating during school events. SNTs tailored their ways of speaking and communicating to fit individual parents to support CNDs through active collaboration. Furthermore, the children were connected to specialists based on their traits and difficulties. Thus, information about CNDs was shared among the teaching staff, and after multidisciplinary and multifaceted discussions on support policies, consistent support approaches were implemented through collaboration based on shared understanding.

## Discussion

This study explored the specific support experiences of SNTs for CND support in mandatory schools. The results showed that SNTs provided various types of support considering the traits of CNDs. The strategies to support CNDs and the role of SNTs are discussed.

SNTs first made efforts to understand the CND’s backgrounds by checking existing surveys and handover materials, sharing information from other teachers and parents, and observing CNDs in their daily lives. It was inferred that by understanding CND’s backgrounds, SNTs recognized their traits and linked them to support CNDs in various situations. For example, SNTs devised explanations and guidance during the health examinations, based on the traits of the CNDs. Studies examining SNTs’ sense of difficulty related to health examinations at school reported “children being afraid of having instruments inserted” as an associated factor, noting that trait-specific approaches, such as “providing advance explanations, guidance, and practice,” are required [16]. Children who are uncomfortable with new situations or have sensory sensitivity may be afraid of an otoscope being inserted into their ears during an ENT examination. Therefore, allowing them to touch the instrument beforehand or engaging in roleplay scenarios that simulate an actual examination may help them find the examination and instrument more familiar and reduce their fear. Without effective guidance or interviews, some CNDs may be unable to undergo health examinations, potentially leading to overlooked health issues. Therefore, tailoring guidance and interviews to their traits is crucial to promote and maintain their health.

Understanding CND’s backgrounds is also considered to be the basis for providing support to reduce difficulties associated with ND traits. Understanding the traits of CNDs is also essential when they panic or when adjusting the environment to take sensory sensitivity into consideration. In addition, to provide support to reduce difficulties associated with ND traits, it is necessary to become someone with whom the children feel safe to engage. Therefore, SNTs acted consciously to become a reassuring and familiar presence and were sincere in not losing the child’s trust. This underscores the importance of maintaining trust, especially for children with dichotomous thinking [17] who may struggle to change their perceptions once formed. SNTs place significant emphasis on first impressions, striving to maintain trust by keeping promises and being honest. Furthermore, the SNTs were receptive and showed an empathic understanding of the CNDs. Studies suggest that rejection from teachers can lead to academic struggles, social exclusion, and peer rejection, particularly for children with ADHD, which in turn can result in lower self-esteem and increased feelings of loneliness [18]. It was inferred that the receptive support of SNTs contributes to the psychological aspect of CNDs, such as reducing their sense of isolation. 

Furthermore, it is clear that SNTs are aware of promoting individual children’s development and adaptation to school life in their daily interactions with CNDs. SNTs emphasize fostering basic communication skills in children, helping them understand appropriate interpersonal distances and consider others’ feelings through daily interactions with SNTs rather than using specialized training. Concurrently, when supporting CNDs, it is presumed that spoiling them and allowing them to do whatever they want will hinder the growth of their social skills. For CNDs who will live within the rules of society in the future, SNTs are thought to set certain rules for their support. Additionally, for children who have difficulty making friends [19], SNTs assist in building friendships through activities conducted in the school infirmary. Studies analyzing the relationship between social support and bullying indicate that support from classmates is the most important means of reducing bullying incidents [20]. Based on this, SNT approaches to support friendship building may serve as measures to mitigate social exclusion within the classroom. Furthermore, individuals with ND have been reported to often have low self-esteem due to feelings of guilt over failures or negative experiences [21]. The SNTs focused on the psychological growth of the CNDs and, among other things, engaged with them in a way that increased their sense of self-esteem. Since low self-esteem in adolescence and young adulthood often leads to mental health problems in adulthood [22], boosting self-esteem and self-worth is crucial for preventing future comorbidities. The receptive, self-esteem-boosting attitudes of SNTs may help reduce mental health problems.

The support described so far was not implemented by SNTs alone. Teachers, parents, specialists, and others also collaborated because if each faculty member were to provide a disparate response, CNDs would become confused, and their sense of trust would be undermined.

Support approaches identified in this study also extend to medical interviews and health examinations. Given that schoolteachers’ communication methods with ASD are frequently specific, objective, and readily adaptable to medical situations [23], the SNT support identified in this study may also be applicable to nurses working in hospitals when dealing with CNDs. Furthermore, surveys of nurses have indicated that they lacked confidence in providing counseling services to children with ASD and their families and that educational programs on this topic should be included in nursing school curricula [24,25].

Although ND traits vary among individuals, and the support approaches identified in this study may not apply to all CNDs, they can serve as valuable resources for students in SNT training institutions. By incorporating these approaches into educational curricula, SNTs may feel better equipped and less daunted by the challenges they may face when they begin working after graduation.

Limitations of the study

This study has several limitations. First, as the survey was conducted in Japan and focused on SNTs, a profession unique to the country, the findings may not be generalizable to other settings or countries. Additionally, the support approaches identified may have been influenced by the individual experiences of participants, underscoring the need for further quantitative research on a broader, nationwide scale.

## Conclusions

SNTs engaged with CNDs in various situations, considering children’s traits and their own duties. Their approaches were summarized as providing support to reduce difficulties associated with ND traits, assessing children’s health status through effective interviews and guidance, promoting individual children’s development and adaptation to school life, becoming a person with whom children feel safe to engage, understanding children’s backgrounds that may not be easily apparent, and collaborating to provide consistent support. To support children’s health and promote their smooth adaptation to school life, these approaches should be implemented as part of daily SNT work. Incorporating the SNT approaches identified in this study into medical practice and school nursing in countries without an SNT system may improve care for CND and enhance their QOL. Further research is warranted to develop evidence-based procedures to provide assistance to CNDs in the future, independent of national differences and individual experiences.

## References

[1] American Psychiatric Association (2013). Diagnostic and Statistical Manual of Mental Disorders (DSM‐5®). https://psychiatryonline.org/doi/book/10.1176/appi.books.9780890425596.

[2] Salari N, Rasoulpoor S, Rasoulpoor S (2022). The global prevalence of autism spectrum disorder: a comprehensive systematic review and meta-analysis. Ital J Pediatr.

[3] Sayal K, Prasad V, Daley D, Ford T, Coghill D (2018). ADHD in children and young people: prevalence, care pathways, and service provision. Lancet Psychiatry.

[4] Maenner MJ, Warren Z, Williams AR (2023). Prevalence and characteristics of autism spectrum disorder among children aged 8 years—autism and developmental disabilities monitoring network, 11 sites, United States, 2020. MMWR Surveill Summ.

[5] Saito M, Hirota T, Sakamoto Y (2020). Prevalence and cumulative incidence of autism spectrum disorders and the patterns of co-occurring neurodevelopmental disorders in a total population sample of 5-year-old children. Mol Autism.

[6] (2023). Ministry of Education, Culture, Sports, Science and Technology. Results of a survey of special education students enrolled in regular classes. https://www.mext.go.jp/content/20230524-mext-tokubetu01-000026255_01.pdf.

[7] Inoue Y, Takehana Y (2019). Yogo teacher support process for children with developmental disabilities at an elementary school. Bull Tokyo Gakugei Univ Divi Arts Sports Sci.

[8] Okada K (2011). The Yogo teacher, the health room, and health education at school in Japan. Asian Perspectives and Evidence on Health Promotion and Education.

[9] Atladóttir HO, Schendel DE, Lauritsen MB, Henriksen TB, Parner ET (2012). Patterns of contact with hospital for children with an autism spectrum disorder: a Danish register-based study. J Autism Dev Disord.

[10] Matsumoto R, Mitsuda T (2019). About coping and duties burden of school nurse: focus on experience (in Japanese). Bull Kagoshima Women's Coll.

[11] Zablotsky B, Bramlett MD, Blumberg SJ (2020). The co-occurrence of autism spectrum disorder in children with ADHD. J Atten Disord.

[12] Kurasawa S, Tateyama K, Iwanaga R, Ohtoshi T, Nakatani K, Yokoi K (2018). The age at diagnosis of autism spectrum disorder in children in Japan. Int J Pediatr.

[13] Kajiya S (2015). The revision of DSM-5 and support for children in the gray zone. Ann Rep Junior Coll.

[14] Guest G, Bunce A, Johnson L (2006). How many interviews are enough? An experiment with data saturation and variability. Field Methods.

[15] Graneheim UH, Lundman B (2004). Qualitative content analysis in nursing research: concepts, procedures and measures to achieve trustworthiness. Nurse Educ Today.

[16] Oe K, Sato Y (2021). The difficulties and coping strategies of school nurses regarding checkups at school for children with developmental disorders in regular class. Yamagata Med J.

[17] Suzuki N, Hirai M (2023). Autistic traits associated with dichotomic thinking mediated by intolerance of uncertainty. Sci Rep.

[18] Plantin Ewe L (2019). ADHD symptoms and the teacher-student relationship: a systematic literature review. Emot Behav Difficulties.

[19] De Boer A, Pijl SJ (2016). The acceptance and rejection of peers with ADHD and ASD in general secondary education. J Educ Res.

[20] Humphrey N, Symes W (2010). Perceptions of social support and experience of bullying among pupils with autistic spectrum disorders in mainstream secondary schools. Eur J Spec Needs Educ.

[21] Capelatto IV, de Lima RF, Ciasca SM, Salgado-Azoni C (2014). Cognitive functions, self-esteem and self-concept of children with attention deficit and hyperactivity disorder. Psicol Reflex Crit.

[22] Trzesniewski KH, Donnellan MB, Moffitt TE, Robins RW, Poulton R, Caspi A (2006). Low self-esteem during adolescence predicts poor health, criminal behavior, and limited economic prospects during adulthood. Dev Psychol.

[23] Arai Y, Okanishi T, Nakamura Y, Maegaki Y (2023). Successful perioperative preparation of a child with autism spectrum disorder in collaboration with his school for special needs education: a case report. Front Psychiatry.

[24] Shawahna R (2021). Self-rated familiarity with autism spectrum disorders among practicing nurses: a cross-sectional study in the palestinian nursing practice. BMC Nurs.

[25] Alabdulaziz HM, Alghamdi SA, Aladwani AM, Alharthi RA, Almadani LK, Niyazi KA, Alharbi YM (2024). Assessment of knowledge and attitudes toward children with autism spectrum disorder among undergraduate nursing students. Cureus.

